# Study on the Pore Structure Characteristics of Ferronickel-Slag-Mixed Ternary-Blended Cement

**DOI:** 10.3390/ma13214863

**Published:** 2020-10-29

**Authors:** Won Jung Cho, Min Jae Kim, Ji Seok Kim

**Affiliations:** 1 Department of Civil and Environmental Engineering, Hanyang University, Ansan 15588, Korea; jiseok@hanyang.ac.kr; 2 Department of Civil, Environmental and Architecture Engineering, Korea University, Seoul 02841, Korea; manta209@korea.ac.kr

**Keywords:** ferronickel slag, blast furnace slag, long term hydration, X-ray tomography

## Abstract

Pore structure development in Portland cement, fly ash, or/and ferronickel slag (FNS) was investigated using mercury intrusion porosimetry and X-ray CT tomography. The progress of hydration was observed using X-ray diffraction (XRD) analysis and compressive strength while durability of concrete was monitored by chloride penetration resistance and chloride profiles. Mercury intrusion porosimetry (MIP) results suggested that the blended cement had a higher porosity while lower critical pore size. The major reason to this increased porosity was the formation of meso and micro pores compared to ordinary Portland cement (OPC). In terms of chloride transport, replaced cement, especially ternary-blended cement had higher resistance to chloride transport and exhibited slightly lower development of compressive strength. X-ray CT tomography shows that the influence of pore structure of ternary-blended cement on the ionic transport was strongly related to the pore connectivity of cement matrix.

## 1. Introduction

Concrete is a nonhomogeneous and porous construction material that includes pore network of different sizes and shapes. Its physical and chemical properties are strongly influenced by this pore structure. For instance, properties such as strength, corrosion resistance, durability, and permeability are closely related to the characteristics of the pore structures for cement-based materials [1]. Therefore, for a better understanding of these characteristics, it is important to study in details the pores present in the concrete matrix. In general, use of mineral admixtures such as fly ash (FA) or ferronickel slag causes grain refinement that leads to reduced permeability [2].

Ferronickel slag (FNS) is an industrial by-product obtained after nickel ore and bituminous coal are melted at high temperatures (between 1500 °C and 1600 °C) and thereafter separated from ferronickel [3,4]. The production of FNS is estimated to be about 2 million tons in Korea [3] and more than 30 million tons in China [5]. Because of the depletion of natural aggregate, there were numerous efforts to use FNS as a replacement of aggregates [3,6]. Furthermore, the utilization of FNS as a binder was also investigated [5,7]. FNS is bound in a crystalline structure known as forsterite (Mg_2_SiO_4_) and its major oxides are SiO_2_, MgO, Fe_2_O_3_, CaO, and Al_2_O_3_. When mixed with ordinary Portland cement (OPC), FNS clinker reacts with Ca(OH)_2_ to create secondary C-S-H gel, thereby contributing to the long-term strength development [8]. However, it has been reported that the development of compressive strength and chloride ion penetration resistance tend to decrease when using FNS as a binder. Some previous research [3,9] showed that mortars with FNS incorporation exhibited similar compressive strength compares to FA.

Recently, it has been reported that the use of FNS has many technical advantages through comparative analysis with conventional cementitious materials as pozzolanic materials, but there is no verification of its reactivity with other existing binders. In that sense, the use of industrial by-products as cementitious materials may reveal different properties compared to conventional materials [10]. Therefore, in order to be safely used, concrete containing any type of by-products should undergo thorough quality control testing, and their properties must be taken into account in the concrete mixture design [11]. Moreover, ternary-blended cement has an advantage of complementing the weakness of each material. In particular, studies on ternary-blended cement using round granulated blast-furnace slag (GGBS) and FA have been completed over the past decades, and are already used as construction materials [12,13]. Therefore, evaluation of FNS ternary-blended cement may enhance the applicability of FNS.

Previous study mentioned above have focused on the investigation of characteristics of FA and FNS as cementitious materials. In order to study the effect of it as an admixture, OPC with binary and ternary-blended mix designs using OPC, FA, or/and FNS were used. The investigation of hydration progress, pore structure refinement, and penetration resistance were also conducted. Since no chemical admixture was used to exclude possible reactions, the experiment was conducted with a specimen that had been cured for a longer period, i.e., one year.

## 2. Experimental Work

### 2.1. Raw Mamerials

**Figure 1:**
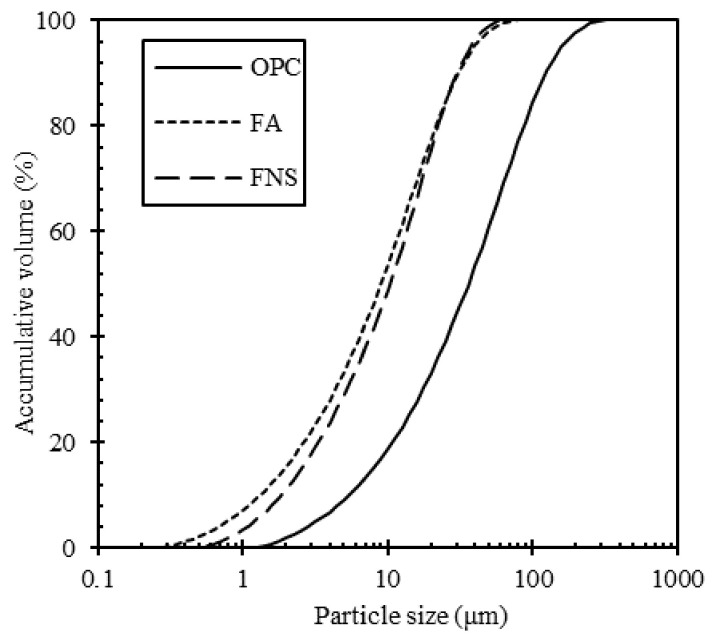
Particle size distribution of ordinary Portland cement (OPC), fly ash (FA), and ferronickel slag (FNS).

The materials used in this study were OPC, FA and electric arc furnace FNS powder produced by Korean company “P.” The particle size distribution is shown in Figure 1. Chemical analysis was carried out with X-ray fluorescence (XRF) and shown in Table 1 together with physical properties. The main chemical components of the FNS powders are SiO_2_, MgO, and Fe_2_O_3_. The CaO contents of FNS was counted for only 6.28% which is lower than OPC but much higher than FA. FA is a by-product generated when using coal as the raw material in thermal power plants. Its chemical composition showed that it is principally composed of silicon with minor amounts of aluminum oxide. Therefore, FA used in this study can be classified as class F, according to ASTM C618 [14].

**Figure 2:**
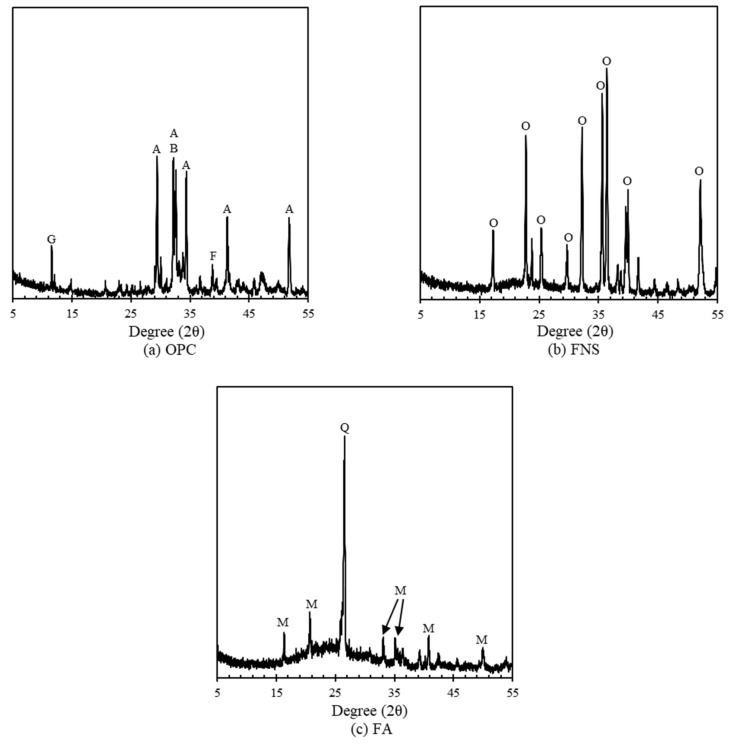
XRD curve of raw materials; (**a**) OPC, (**b**) FNS, (**c**) FA. A: Alite (C_3_S), B: Belite (C_2_S), F: Ferrite (C_4_AF), G: Gypsum (CaSO_4_), O: Olivine (Mg, Fe)_2_SiO_4_Q: Quartz (SiO_2_), M: Mullite (Al_2_Si_6_O_13_).

The Figure 2 presented the X-ray diffraction (XRD) curves of the OPC, FA, and FNS, respectively. In the case of OPC, alite (C3S, 3CaO∙SiO_2_) and belite (C2S, 2CaO∙SiO_2_) are the main clinker while other clinkers such as gypsum, periclase, brownmillerite (4CaO∙Al_2_O_3_∙Fe_2_O_3_) accounted for lower quantities. For FA, XRD patterns mostly consisted of high silica contents, while for FNS the composition mainly accounted for forsterite (Mg_2_SiO_4_) and fayalite (Fe_2_SiO_4_) which have crystalline nature. Most of the MgO peaks detected from XRF analysis indicated forsterite and it originally shows very late hydration which takes about 2 years for complete reaction [15].

### 2.2. Mix Proportion

The mix proportions of the specimens are shown in Table 2. The 100% plain cement was selected as a reference while replacement ratio of 30% (by mass) was chosen for each test specimen. Although, American Concrete Institute (ACI) recommends that a maximum replacement rate of Class F FA should be 25%, for the sake of a process of calculating the mix proportion of ternary-blended, the maximum replacement ratio is set to 30% in this study. For fine aggregates, washed aggregate with specific gravity of 2.60 g/cm^3^ and fineness modulus of 2.73 was used. In the case of gravel, crushed aggregate with specific gravity of 2.62 g/cm^3^ and maximum size of 25 mm was employed.

### 2.3. Specimen Preparation and Test Method

In this study, the hydration behavior and properties of ternary blends incorporating FNS and conventional binders are evaluated. For this, paste specimens were used in accordance with X-ray diffraction pattern (XRD) and mercury intrusion porosimetry (MIP) tests while concrete was used for compressive strength, rapid chloride ion permeation tests (RCPT), and water permeability to observe pore structure characteristics in terms of durability. The prepared specimens were cured up to 365 days, because there was no addition of chemical admixtures to eliminate compound effects on the cement matrix. Except for the compressive strength test, the experiments were conducted by a single specimen or samples without replications. Table 2 summarizes the experimental outline.

#### 2.3.1. X-ray Diffraction (XRD)

In order to examine the hydration products, cement paste specimens were mixed with 0.45 of a total water-binder ratio (W/B). After mixing of the dry powder with distilled water for 5 min, the pastes were placed into a cubic mold (50 × 50 × 10 mm), which was in turn cured at room conditions (20 ± 2 °C, RH 65 ± 5%) for 24 h. Then, the specimens were demolded and stored in a water bath (20 ± 2 °C) for specified periods. After it, the hardened paste specimens were fragmented or ground off for its application to each microscopic examination. To mineralogically determine hydration products, XRD analysis using MDI JADE package of software [16] was employed. The scanning was carried out by using D/Max-2500 diffractometer (Rigaku Corporation, Shoshima, Tokyo, Japan.) in the diffraction range (2θ) of 5–60° at a rate of 4.0°/min with 40 kV voltage and 100 mA current with a wavelength of l.5405 A° (1 A° = 0.1 nm).

#### 2.3.2. Mercury Intrusion Porosimetry (MIP) 

To investigate the porosity and pore size distribution of the paste samples, mercury intrusion porosimetry was applied by using AutoPore IV (Micromeritics Instrument Corporation, Norcross, Atlante, GA, USA). At the age of curing, crushed paste samples were placed in acetone to stop further hydration and then dried to remove residual water in an oven at 50 °C for 24 h. Then, the scraps were placed in a tube filled with mercury. The obtained samples were initially evacuated to about 50 µm mercury and the low pressure was generated up to 0.20 MPa by nitrogen gas. The specimen was subsequently intruded by mercury at a filling pressure ranging from 0.0037 to 413 MPa and the mercury intrusion volume was recorded at each corresponding pressure. The pore diameter was derived from the pressure using the Washburn‘s equation as given in Equation (1) [17].

(1) $$d\;=\frac{-4\gamma \cos\theta }{P}$$

where, d is the pore diameter (cm); γ is the surface tension (N/cm); P is the pressure (MPa); and θ is the contact angle (°) in which the present study took this value as 130°.

#### 2.3.3. Compressive Strength

Cylindrical concrete specimens (Ø100 × 200 mm) were fabricated according to ASTM C192 [18] and cured at room conditions (20 ± 2 °C, RH 65 ± 5%) for 365 days. Compressive strength was measured at 365 days of curing following the standard ASTM C39 [19]. The replication was three and average of them was presented in this paper.

#### 2.3.4. Rapid Chloride Penetration Test (RCPT)

The RCPT was carried out in accordance with ASTM C1202 [20]. Cylindrical concrete specimens (Ø100 × 200 mm) were cured under standard curing conditions until testing. At the age of 365 days, concrete samples were cut to 50 mm thick slices and 100 mm nominal diameter and kept in air for 1 h for surface dry. Then, they were brushed for rapid setting coating onto the side surface and placed in room temperature to ensure complete side isolation. After that, samples were kept in a vacuum desiccator for 3 h with 50 mm Hg pressure followed by 18 h of immersion in water. Water was removed from the saturated specimens, it could then be subjected to a potential difference of 60 V and direct current for 6 h at 20 °C environmental temperature. Test samples were exposed at one side of the specimen in contact with 3% sodium chloride (NaCl) solution and the other in contact with 0.3 M of sodium hydroxide (NaOH) solution. Charged current was calculated by Equation (2) as shown in below.

(2) $$Q=I_{0}+2\cdot I_{30}+2\cdot I_{60}+2\cdot I_{90}+\cdot \cdot \cdot +2\cdot I_{360}\;\cdot$$

where, Q is the total charge passed (C); $I_{0}$ is the initial current immediately after voltage is applied (mA), and $I_{t}$ is the current at t min after voltage applied (mA).

#### 2.3.5. Chloride Profile

The resistance of chloride transport in concrete was evaluated by measuring the chloride profile using cylindrical concrete specimens (Ø100 × 50 mm). One end for each specimen was coated by epoxy resin, and the rest of the surface was uncoated to induce one-direction penetration of chloride ions through concrete. Then, the specimens were immersed in a 4 M NaCl solution for 365 days. Samples were ground from the surface with 5.0-mm increments using a diamond blade grinder for chloride profiling. The collected dust sample was diluted in a nitric acid solution to extract chloride ions; then the diluted solution was taken to measure the concentration of chloride ions by cover depth using an ion-selective electrode. Once the chloride profile was obtained, the diffusion coefficient and surface chloride concentration were determined by solving Fick’s 2nd law as given by

(3) $$C\left(x,t\right)=C_{S}\left(1-\mathrm{erf}\frac{x}{2\sqrt{D\cdot t}}\right)$$

where, C(x, t) is the chloride concentration at depth x after time t (s), C_S_ is the surface chloride concentration (%), x is the depth (m), D is the apparent diffusion coefficient (m^2^/s), and t is the time of exposure (s).

#### 2.3.6. X-ray CT Imaging

After curing for 365 days, the concrete specimens (Ø100 × 200 mm) were cut to 50 mm in height and 100 mm in diameter and kept in ambient temperature for 1 h stabilization of mass. Then, samples were placed in CT scanning. The details of equipment and operation are given in Table 3.

After scanning, the obtained images are filtered to remove the noises and segment the pores using OTSU (otsu algorithm) [21]. Then, reconstruct the obtained images into a 3D image in Avizo software [22]. Detailed scan conditions are described in Table 4.

## 3. Results and Discussion

### 3.1. Hydration Products

**Figure 3:**
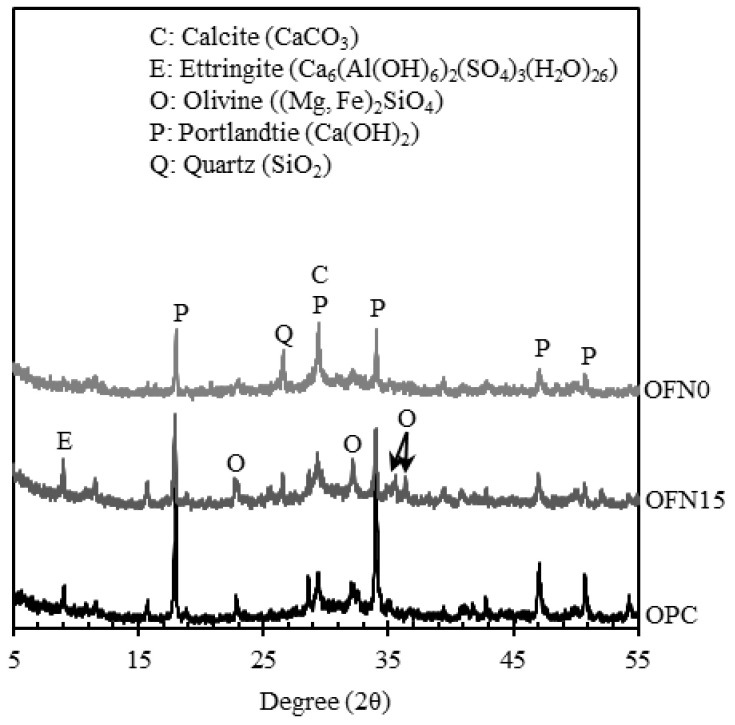
XRD curve of paste for 365 days of curing.

To determine the hydraulic reactivity of ternary-blended cement containing OPC, FNS, and FA, the XRD examination was performed for pastes at 365 days, as given in Figure 3. Hydration products for OPC as control mainly include portlandite (Ca(OH)_2_) and ettringite (Ca_6_Al_2_(SO_4_)_3_∙32H_2_O). As for the mix of OPC with FA (OFN0), hydration products mainly consisted of portlandite, calcite (CaCO_3_), and unreacted particles (i.e., quartz; SiO_2_), while OFN15 was indicative of the presence of identical hydration products except for anhydrate olivine crystalline ((Mg, Fe)_2_∙SiO_4_), presumably being originated from raw FNS powder. In fact, it is notable that there was no further formation of hydration products in FNS-mixed ternary-blended cement paste. A formation of calcite in OPC, OFN0, and OFN15 may be attributed to long-term curing age. A reduction of the peak intensity for portlandite was observed in the XRD curves for OFN15 and OFN0 paste unlike OPC, reflecting that a partial replacement of supplementary cementitious materials in binder would be more likely to form C-S-H gel, together with the depletion of CH in the cement matrix.

### 3.2. Pore Structure Measurement of Paste

**Figure 4:**
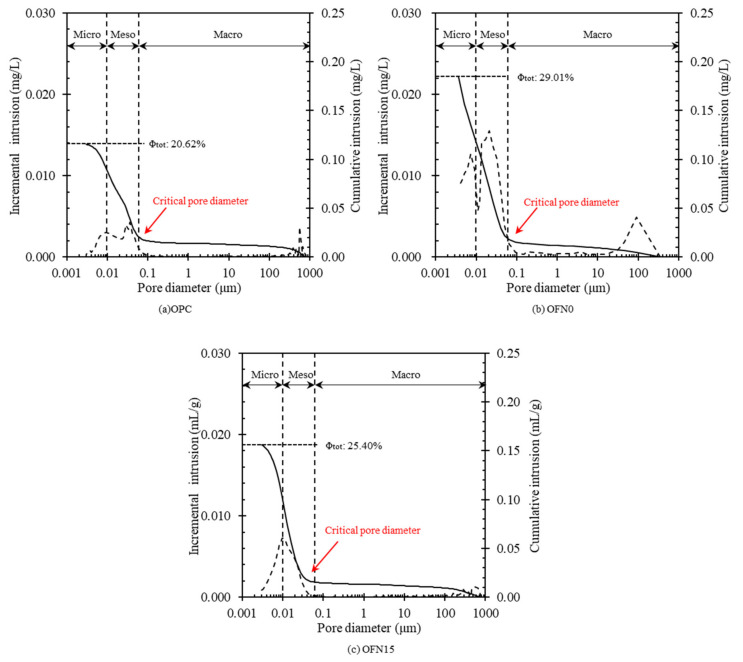
Pore distribution of paste for 365 days of curing; (**a**) OPC, (**b**) OFN0, (**c**) OFN15.

To characterize the pore structure of FNS-mixed ternary-blended concrete, the pore distribution was preliminarily examined by MIP. Paste was used for the MIP to allow the pores to be formed in the cement matrix rather than at the interfacial zones between paste and aggregate; thus, the impact of distribution of aggregate was removed. Figure 4 gives the information of the pore structure from the MIP test results for each mix. When it comes to the porosity, OPC had the lowest value accounting for 20.62%, while OFN0 produced the highest porosity about 29.01%. For OFN15, 25.40% of total porosity was observed at 365 days. It suggests that total porosity is much influenced by cementitious materials; in fact, a decrease in the content of cement resulted in an increase in the porosity, presumably because of the refinement of the pore structure with degree of hydration. In turn, the decreased critical pore diameter was also detected in blended cement. In particular, OFN15 had the lowest size of critical pore diameter, accounting for 0.039 µm, while OPC and OFN0 had 0.081 and 0.077 µm, respectively. It has an important implication in that the lower pore diameter could provide limited paths for ions and molecules to be mobile in OFN15 concrete. As for the pore distribution, OPC consisted of significantly lower volume of micro and meso pores. A sharp rise of the volume for micro and meso pores were observed in OFN0 and OFN15 with different margin in macro pore volumes.

### 3.3. Compressive Strength

**Figure 5:**
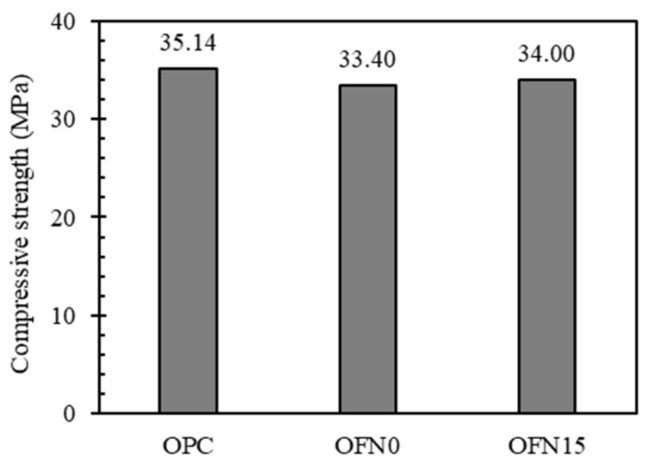
Compressive strength of concrete.

Fundamental properties of ternary-blended cement containing FA and FNS were investigated in terms of compressive strength as given in Figure 5. The development of compressive strength was dependent on mix types. OPC was indicative of the highest compressive strength accounting for 35.14 MPa at 365 days, while OFN0 and OFN15 achieved 33.40 and 34.00 MPa, respectively. A slightly decrease in the compressive strength for OFN0 and OFN15 in a long term may be, as expected, attributed to a latent hydration process. It is notable that OFN15 exhibited increased strength value compared to OFN0, implying that some factors make OFN15 mix more feasible in situ by enhancing the strength development, such as the reduction of a free water to clinker ratio induced by lower reactivity of FNS. Despite the lower level of compressive strength for OFN0 and OFN15, it seems that the gap compared to OPC is not much considerable.

### 3.4. Chloride Transport

**Figure 6:**
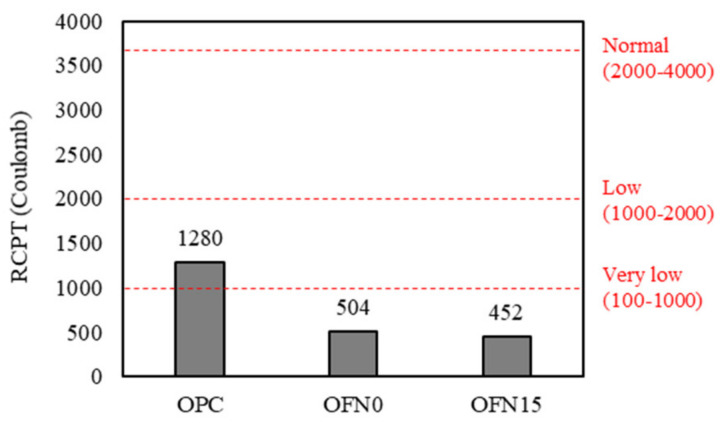
Chloride penetration resistance of concrete.

The charge passed through concrete specimen is given in Figure 6. It is evident that OPC ranked the highest level for chloride ion penetrability, while OFN0 and OFN15 imposed very low levels at an identical experiment. Irrespective of mix type, an increase incorporation of cementitious materials resulted in a decrease in the charge passed. For instance, OPC had 1280 Coulombs while OFN0 and OFN15 had a reduction of charge passed in a similar range, accounting for 452 and 504 Coulombs, which is indicative of “Very Low” range for chloride ion penetrability according to the ASTM C 1202 [20]. FA in the concrete mix reduced the critical pore size, implying that the pore structure in OFN0 and OFN15 was mainly governed by small capillary pore or/and gel pore, thereby leading to reduced ionic penetrability. However, the total porosity for OFN0 and OFN15 was even higher than OPC control as seen above. It suggests that ionic transport in concrete is much influenced by pore distribution rather than pore volume [23]; the higher volume for micro and gel pores may be beneficial in mitigating ionic transport at a given total porosity. It is notable that the charge passed for OFN15 was slightly lower in general than for OFN0, presumably because of the further formation of micropores (i.e., gel pores).

**Figure 7:**
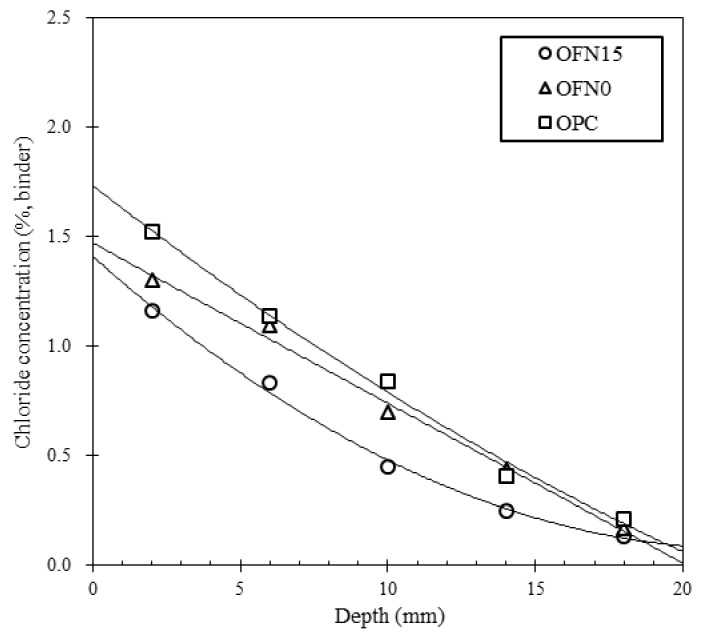
Chloride profile at each depth.

The resistance of concrete against ionic/molecular transport was evaluated by chloride profiling of concrete which was submerged in saltwater. Because chloride transport was driven by only one directional diffusion, the apparent diffusion coefficient and surface chloride concentration were simultaneously determined by using chloride ingresses at different depths for a certain duration of immersion. As seen in Figure 7, the concentration of chloride was reduced with the depth of cover concrete, irrespective of whether or not the cementitious materials were incorporated. In particular, the chloride ingress was mostly nullified at 20.0 mm from the concrete surface for all specimens because of the dense pore structure of concrete in long-term curing age. The replacement of cement by binder showed benefits in lowering the chloride ingress at all depths, resulting from a pore refinement of concrete thus a reduced diffusivity of chloride ions. For OFN15, there was a reduction of the surface chloride, accounting for 1.32%, while OFN0 and OPC presented a higher range of about 1.30 and 1.52%. The diffusion coefficient for OPC, OFN0, and OFN15 was 1.92 × 10 ^−11^, 1.78 × 10^−11^, and 1.39 × 10^−11^ m^2^/s, respectively. The further reduced value of the diffusion coefficient for OFN15 may arise from unreacted crystalline FNS clinkers, which leads to pore refinement (i.e., pore blocking), and lower diffusion coefficient. In fact, FA was effective in pore refinement, leading to a dense pore structure, but only a marginal reduction of the chloride diffusivity was achieved. However, incorporation of FNS had an effect of the pore-blocking in inner pores, so that the chloride ingress was decreased at the inner depth (from 10–15 mm).

### 3.5. X-ray Tomography

**Figure 8:**
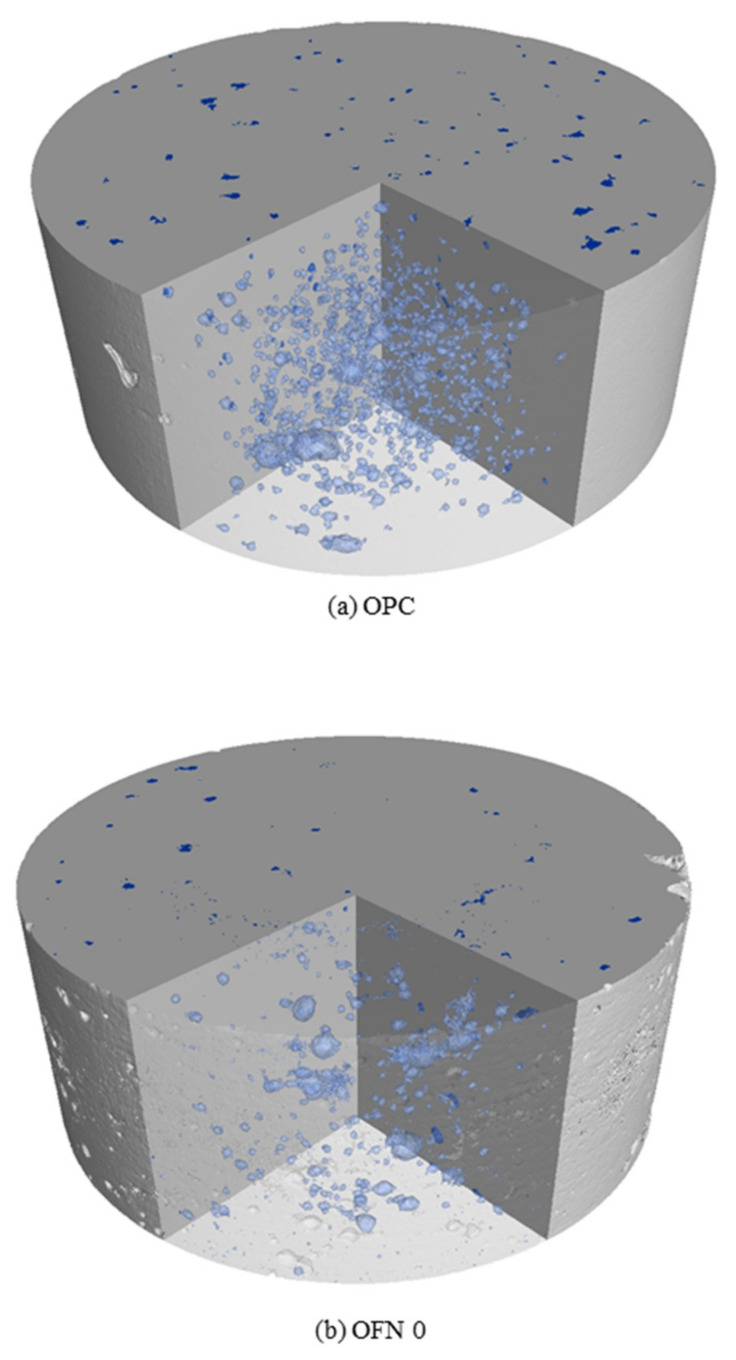

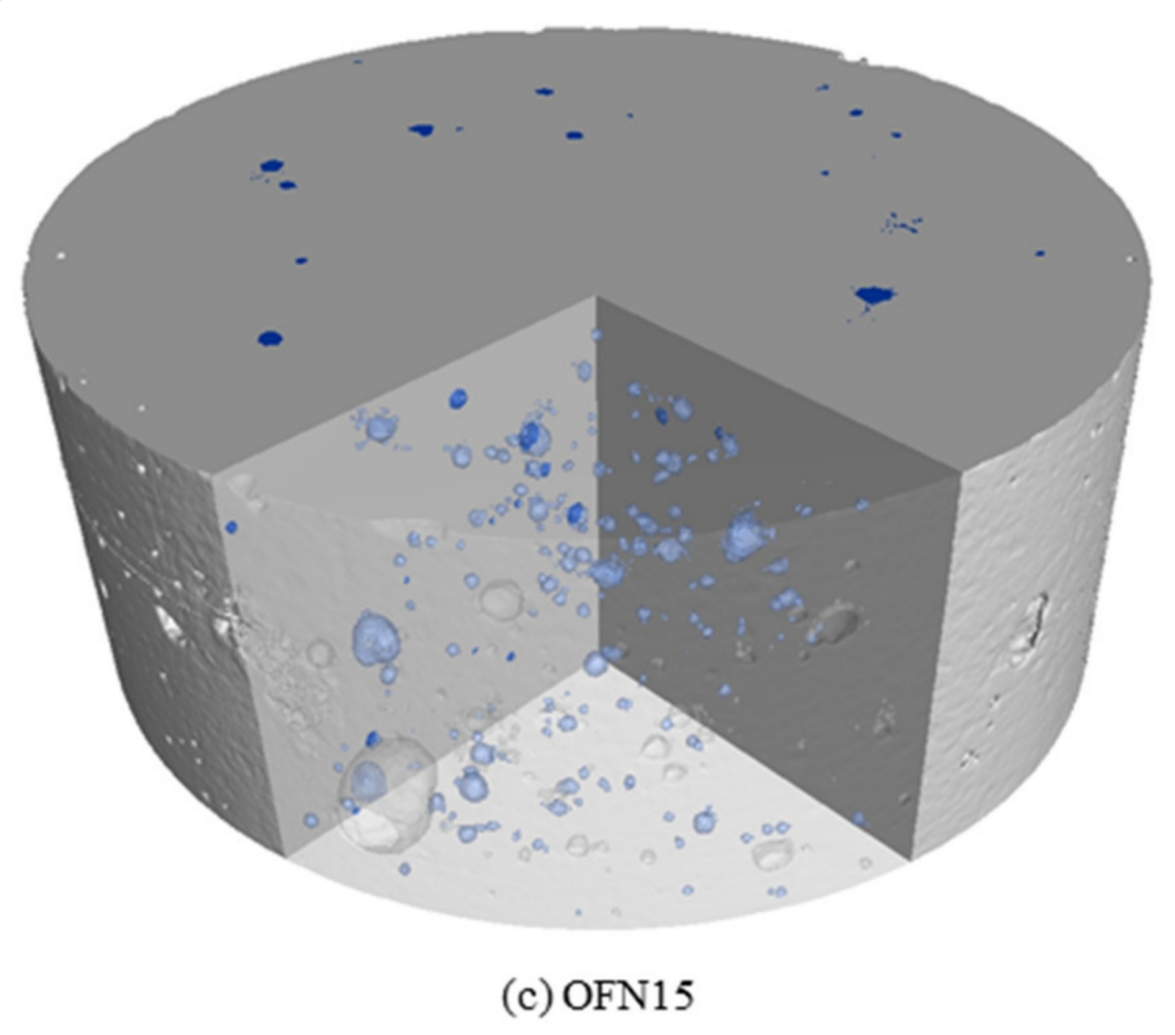
X-ray tomography of concrete; (**a**) OPC, (**b**) OFN0, (**c**) OFN15.

Figure 8 represents the 3D pore size distribution in concrete distinguished by blue color. For OPC, the average porosity is 1.31% while OFN0 and OFN15 showed 0.75 and 0.56, respectively. This result indicates that the porosity variation derived by X-ray tomography test is quite different compared to MIP test results. The measurement of sample by MIP analysis used paste to eliminate the effect of aggregate, while X-ray tomography test specimen was concrete. Thus, the differences of test results between X-ray tomography and MIP test may arise by the interface between aggregate and cement matrix.

The smallest pore diameter is mainly detected in OPC concrete, however, macro pores that are not interconnected were detected in OFN15. The distribution of smallest pore was relatively detected in OFN0, but interrelated network was also detected. As seen in Figure 6 and Figure 7, the OPC and OFN0 produced the higher rate of chloride transport in terms of diffusion coefficient compared to OFN15. This may be attributed to the connectivity of pores, thus chloride ions penetrated in pore paths, though lower volume of macro pores. Compared to OFN15, OFN0 showed vulnerable results to ionic transport, however, due to enhanced pore structure, it showed better resistance than OPC. Furthermore, the distribution of pore was affected by FNS; low reactivity of FNS increased water to clinker ratio then resulted in increased formation of C-S-H gel, thus a dense pore structure that is not connected thus may block the ionic transport [24].Thus the pore structure of concrete would be refined with incorporation of FNS as a binder. The pore area of the C-S-H gel was not in the range that could be detected by X-ray CT, thus it was not obtained.

## 4. Conclusions

In this study, the pore structure characteristics of blended cement were investigated by microscopic analysis (XRD and MIP analysis), compressive strength, chloride transport and X-ray CT. Detailed conclusions from this investigation are as follows:

(a) From XRD analysis, a weakened intensity of portlandite peak was observed in blended cement compared to OPC because of the pozzolanic reaction in the cement mix. When it comes to the porosity, due to the pore refinement of FA or/and FNS, blended cement showed an increased porosity while decreased critical pore diameter compared to OPC. An incorporation of FA or/and FNS as a binder in concrete resulted in decreased compressive strength compared to OPC concrete, however, the difference was not significant.
(b) As for the resistance to chloride penetration, the OPC concrete indicated higher level of chloride penetration while the concrete containing other binder was equally ranged in “very low” range.
(c) The diffusion coefficient and concentration of concrete were obtained by profiling of chloride concentration at each depth after immersed in salt solution. The surface chloride of OPC, OFN0, and OFN15 ranged from 1.30 to 1.52% while the diffusion coefficient was in the range of 1.39–1.92 × 10^−11^.
(d) The pore distribution of concrete measured by X-ray CT analysis indicated that pore connectivity marginally decreased because of the incorporation of binder. The apparent pore refinement was detected in OFN15 derived from increased amount of C-S-H gel, which was attributed to lower reactivity of FNS induced by increased water to clinker ratio.

## Figures and Tables

**Table 1:** Chemical and physical properties of raw materials.

	Oxides (%)							Physics		Loss on Ignition (%)
	CaO	SiO_2_	Al_2_O_3_	MgO	Fe_2_O_3_	SO_3_	TiO_2_	Density (g/cm^3^)	Fineness (cm^2^/g)	
OPC	66.98	17.43	3.97	1.60	4.16	3.41	0.27	3.14	3.539	0.40
FA	3.93	65.48	18.48	0.64	5.81	0.80	1.12	2.2	3.850	1.95
FNS	6.28	48.23	3.59	23.01	15.76	0.50	0.11	3.14	3.400	0.02

**Table 2:** Mix proportion and experimental outline.

Type		Mix Proportion (g/m^3^)						Experiment Outline
		OPC	FA	FNS	Sand	Gravel	W/B	
Paste	**OPC**	1301.3	-	-	-	-	0.45	XRD MIP
	OFN15	887.1	375.0	-	-	-	0.45	
	OFN0	893.5	191.5	191.5	-	-	0.45	
Concrete	OPC	360.4	-	-	756.9	1131.7	0.45	Compressive strength Rapid chloride penetration depth Chloride profile X-ray CT image
	OFN0	249.5	106.9	-	748.4	1119.0	0.45	
	OFN15	250.9	53.8	53.8	161.3	752.6	0.45	

**Table 3:** Equipment specification.

X-ray Tube	Tube Type	Target Design	High Voltage Range	Tube Current Range	Max. Tube Power/Target Power	Focal Ppot Size	Min. FOD	Standard Filament
MXR-320HP/11AX	Closed steel tube	Bipolar	30–320 kV	0.01–2 mA	800 W/1800 W	0.4 mm	25 mm	tungsten

**Table 4:** Scan condition.

Diameter (mm)	Voltage (kVp)	Current (mA)	Transmission Time (sec)	Source-Object Distance (mm)	Pixel Pitch (mm)
100	200	0.8	1	316	0.106488

## References

[1] Živica V. (1997). Relationship between pore structure and permeability of hardened cement mortars: On the choice of effective pore structure parameter. Cem. Concr. Res..

[2] Mehta P.K. (1989). Pozzolanic and cementitious by-products in concrete—Another look. Spec. Publ..

[3] Cho B.S., Kim Y.U., Kim D.B., Choi S.J. (2018). Effect of ferronickel slag powder on microhydration heat, flow, compressive strength, and drying shrinkage of mortar. Adv. Civil. Eng..

[4] Choi Y.C., Seongcheol C. (2015). Alkali–silica reactivity of cementitious materials using ferro-nickel slag fine aggregates produced in different cooling conditions. Constr. Build. Mater..

[5] Huang Y., Qiang W., Mengxiao S. (2017). Characteristics and reactivity of ferronickel slag powder. Constr. Build. Mater..

[6] Sakoi Y., Aba M., Tsukinaga Y., Nagataki S. Properties of concrete used in ferronickel slag aggregate. Proceedings of the 3rd International Conference on Sustainable Construction Materials and Technologies.

[7] KoNubu K., Shoya M. (1994). Guidelines for construction using Ferronickel slag fine aggregate concrete. Concr. Libr. JSCE.

[8] Lemonis N., Tsakiridis P.E., Katsiotis N.S., Antiohos S., Papageorgiou D., Katsiotis M.S., Beazi-Katsioti M. (2015). Hydration study of ternary blended cements containing ferronickel slag and natural pozzolan. Constr. Build. Mater..

[9] Rahman M.A., Sarker P.K., Shaikh F.U.K., Saha A.K. (2017). Soundness and compressive strength of Portland cement blended with ground granulated ferronickel slag. Constr. Build. Mater..

[10] Qi A., Liu X., Wang Z., Chen Z. (2020). Mechanical properties of the concrete containing ferronickel slag and blast furnace slag powder. Constr. Build. Mater..

[11] Papayianni I., Anastasiou E. (2010). Production of high-strength concrete using high volume of industrial by-products. Constr. Build. Mater..

[12] Cheah C.B., Tiong L.L., Ng E.P., Oo C.W. (2019). The engineering performance of concrete containing high volume of ground granulated blast furnace slag and pulverized fly ash with polycarboxylate-based superplasticizer. Constr. Build. Mater..

[13] Ng P.G., Cheah C.B., Ng E.P., Oo C.W., Leow K.H. (2020). The influence of main and side chain densities of PCE superplasticizer on engineering properties and microstructure development of slag and fly ash ternary blended cement concrete. Constr. Build. Mater..

[14] ASTM C618 (2002). Standard Specification for Coal Fly Ash and Raw or Calcined Natural Pozzolan for Use as a Mineral Admixture in Portland Cement Concrete.

[15] Bernard E., Lothenbach B., Rentsch D., Pochard I., Dauzeres A. (2017). Formation of magnesium silicate hydrates (M-S-H). Phys. Chem. Earth..

[16] Jade (MDI) (1994). Software for X-ray Diffraction Pattern Processing.

[17] Washburn E.W. (1921). Note on a method of determining the distribution of pore sizes in a porous material. Proc. Nat. Acad. Sci. USA.

[18] ASTM C192/C192M (2007). Standard Practice for Making and Curing Concrete Test Specimens in the Laboratory.

[19] ASTM C09 (2014). Standard Test Method for Compressive Strength of Cylindrical Concrete Specimens.

[20] ASTM C1202-18 (2012). Standard Test Method for Electrical Indication of Concrete’s Ability to Resist Chloride Ion Penetration.

[21] Vala H.J., Baxi A. (2013). A review on Otsu image segmentation algorithm. Int. J. Adv. Res. Comput. Eng. Tehnol. (IJARCET).

[22] Scientific T.F. (2018). Amira-Avizo Software.

[23] Kim H., Lee C.H., Ann K.Y. (2019). Feasibility of ferronickel slag powder for cementitious binder in concrete mix. Constr. Build. Mater..

[24] Glass G.K., Buenfeld N.R. (2000). Chloride-induced corrosion of steel in concrete. Struc. Eng. Mater..

